# How do neurorehabilitation patients perceive their fundamental care needs? Findings from a qualitative study

**DOI:** 10.1002/pmrj.70236

**Published:** 2026-10-15

**Authors:** Carlo Alberto Camuccio, Luisa Cacciante, Enrico Dal Pozzo, Francesca Burgio, Orejeta Diamanti, Mayra Veronese, Matteo Danielis

**Affiliations:** ^1^ Immunology and Molecular Oncology Diagnostics Unit, Veneto Institute of Oncology Padova Italy; ^2^ Laboratory of Healthcare Innovation Technologies, IRCCS San Camillo Hospital Venice Italy; ^3^ IRCCS San Camillo Hospital Venice Italy; ^4^ Neuropsychology Laboratory, IRCCS San Camillo Hospital Venice Italy; ^5^ Department of Biomedicine and Prevention University of Rome Tor Vergata Rome Italy; ^6^ Laboratory of Studies & Evidence‐Based Nursing, Department of Cardiac Thoracic, Vascular Sciences and Public Health, University of Padova Padova Italy

## Abstract

**Background:**

The Fundamentals of Care (FoC) framework provides a structured model to support the delivery of person‐centered nursing care by integrating physical, psychosocial, and relational dimensions. Although the framework has been widely applied in acute care, little is known about its relevance in long‐term neurorehabilitation settings, where patients often face complex, prolonged, and multidimensional care needs.

**Objective:**

To explore how individuals with neurological disorders perceive their fundamental care needs during inpatient rehabilitation.

**Design:**

A qualitative secondary analysis.

**Setting:**

A rehabilitation hospital in Italy with a total capacity of 109 beds.

**Participants:**

We recruited individuals aged 18 years and older with a diagnosis of stroke, multiple sclerosis, or Parkinson disease, or who had undergone neurosurgical intervention for oncological conditions.

**Methods:**

A deductive content analysis was performed, using the FoC framework to guide coding and thematic development.

**Findings:**

The analysis identified care experiences across the three FoC dimensions. In the physical dimension, patients valued hygiene, toileting, mobility, and comfort not only as technical tasks but also as meaningful acts that contributed to dignity and recovery. The psychosocial dimension emphasized the importance of respectful communication, emotional support, privacy, and being informed, factors that fostered agency and trust. In the relational dimension, patients highlighted empathy, continuity, and presence as central to feeling emotionally safe and supported. Health care assistants emerged as key figures in delivering comfort‐focused and relational care. Across all dimensions, fundamental care was perceived as deeply interconnected and resistant to rigid, task‐based models.

**Conclusions:**

This study underscores the value of the FoC framework in capturing the multidimensional nature of care as experienced by neurorehabilitation patients. The findings reinforce the need for nurses and assistants to provide care that extends beyond physical tasks to include relational presence and person‐centered responsiveness.

## INTRODUCTION

Over the past decades, increasing attention has been paid to the quality and safety of nursing care, particularly regarding patients' fundamental needs.^1^ In response to growing concerns about the neglect of these needs in modern health care systems, the Fundamentals of Care (FoC) framework was developed.^2^, ^3^, ^4^ This conceptual model defines the key elements of high‐quality fundamental care and outlines how it can be delivered consistently in a person‐centered, evidence‐based manner.^5^ Its development was informed by both empirical evidence and nursing theory, and was prompted by health care reports highlighting persistent gaps in meeting patients' essential care needs.^6^

The FoC framework identifies three core dimensions that underpin the quality of fundamental care: the nurse–patient relationship, the integration of physical and psychosocial needs, and the contextual factors that influence care delivery.^3^, ^7^, ^8^ At its core, the model emphasizes that effective fundamental care requires a therapeutic relationship built on trust and empathy, through which nurses can address patients' needs—ranging from hygiene, mobility, and nutrition to dignity, privacy, and emotional support.^3^ These needs are not fulfilled in isolation but are shaped and mediated by the relational actions of nurses within specific health care contexts.^2^, ^4^

However, in contemporary, fast‐paced clinical environments, there is a growing concern that technical efficiency and task‐oriented care are being prioritized at the expense of comprehensive and relational care.^6^, ^9^ Fundamental care is often undervalued, perceived as routine or “common sense” work that can be delegated, rather than recognized for its complexity and impact.^10^, ^11^ Much of fundamental care remains undocumented in clinical records, as many of its components are not routinely captured by standard documentation practices.^12^ These elements, often referred to as forms of “invisible” care, include activities like providing psychological support, fostering therapeutic relationships, evaluating care outcomes, and engaging with families, actions crucial to quality care but difficult to observe, measure, or record.^12^, ^13^ Furthermore, nursing interventions are unlikely to be fully effective without the active involvement of patients, because high‐quality fundamental care depends not only on nurses' relational and contextual responsiveness but also on patients' co‐participation in shaping their own care experiences.^14^

Despite its foundational role in promoting health, well‐being, and quality of life, fundamental care remains underresearched in clinical settings. Existing studies have largely focused on its physical aspects, often neglecting relational, psychosocial, and patient‐involvement dimensions.^15^ Evidence nevertheless shows that when fundamental care is delivered effectively, it contributes to higher patient satisfaction, reduced mortality, and improved recovery experiences.^1^, ^16^, ^17^, ^18^ Two recent scoping reviews^15^, ^19^ indicated that the application of the FoC has been predominantly explored in acute care settings—such as medical, surgical, geriatric wards, and emergency departments—whereas comparatively less attention has been given to its implementation in long‐term care, community contexts, or among individuals with chronic conditions.

This highlights the need to extend FoC research and practice to diverse patient groups and care trajectories, including those with complex, prolonged, and multidimensional needs often seen in neurological disorders.^20^, ^21^ To our knowledge, only one study has explored the experience of patients with neurological conditions in relation to the FoC framework, focusing specifically on survivors of stroke.^4^ In this qualitative study, Kitson et al.,^4^ examined 15 stroke survivors during their hospital stay. The findings revealed that patients retained vivid, and often distressing, memories of fundamental care activities such as hygiene, nutrition, mobility, and toileting. Each physical need was closely related to psychosocial and emotional dimensions, including loss of dignity, humiliation, and threats to personal identity. By contrast, the few positive recollections were associated with care episodes in which physical, relational, and psychological needs were addressed in an integrated, person‐centered manner.

Understanding how patients with neurological conditions perceive their fundamental care needs, and how these are addressed in practice, is critical to ensuring high‐quality, person‐centered care across settings.

## METHODS

### Study aims

The purpose of this study is to explore and describe the perceived care needs of individuals with neurological disorders through the lens of the FoC framework.

### Design

This study employed a qualitative secondary analysis (QSA). QSA offers a valuable alternative to conducting redundant primary studies and allows for the efficient use of existing datasets.^22^ According to Heaton's QSA typology,^23^ this study can be categorized as a supra‐analysis, which involves reexamining preexisting data through a theoretical lens that was not part of the original research objectives. In supra‐analysis, the terms and analytic boundaries of the primary study from which the data were derived are intentionally transcended.^23^ Rather than redescribing or aggregating the original findings, supra‐analysis enables the investigation of new theoretical questions that were not the focus of the primary analysis. In this study, we reexamined the data using the FoC framework. Although the framework was not part of the original study design, the richness of the qualitative data allowed for a theory‐informed reinterpretation, generating higher‐level conceptual insights while staying true to participants' original accounts.

The original study explored the experiences of care and hospitalization from the perspective of individuals undergoing neurorehabilitation. Building on this dataset, the present study constitutes a secondary qualitative analysis aimed at examining the patient experience of care, with a specific focus on how individuals with neurological disorders perceived their fundamental care needs during the rehabilitation period. For the purpose of the present analysis, the research question was: How do individuals with neurological disorders perceive their fundamental care needs during the rehabilitation period?

To ensure transparency the research followed the Consolidated Criteria for Reporting Qualitative Research developed by Tong et al.^24^

### Study setting and sampling

Data for this secondary analysis were drawn from a set of narrative interviews conducted with individuals hospitalized in a long‐term neurorehabilitation hospital in the Veneto region of northern Italy. At the time this study (2024), the rehabilitation hospital had a total capacity of 109 beds. Patients had a mean age of 63.4 years and an average length of stay of 82 days. Care was provided 24/7 by registered nurses (RNs) and health care assistants (Table S1), supported by a multidisciplinary team that included physicians, physiotherapists, speech and language therapists, and psychologists.

Participants were recruited according to the following inclusion criteria: (1) diagnosis of stroke, multiple sclerosis, Parkinson disease, or who had undergone neurosurgical intervention for oncological conditions; (2) ability to provide informed consent; (3) ability to understand, speak, and read Italian; (4) hospitalization for at least 21 days, allowing for an initial assessment during the first week followed by integration into the rehabilitation program, giving patients sufficient time to acclimate to the hospital environment; and (5) discharge not scheduled within the following few days (ie, not scheduled within the first 21 days of admission).

Exclusion criteria were as follows: (1) inability to provide valid informed consent; (2) age under 18 years; (3) significant cognitive impairment preventing effective participation in an interview; (4) diagnosed psychiatric disorders; and (5) hospitalization for conditions not requiring neurorehabilitation.

### Data collection

The interviews were conducted in 2024 by two researchers (C.A.C., E.D.P.) with extensive experience in qualitative methods. Both researchers are male. C.A.C. is a nurse with clinical experience in neurological care and experience in qualitative research, and E.D.P. is an anthropologist specializing in phenomenological qualitative studies. One of the authors of the current study served as the principal investigator of the original study and was directly involved in data collection.

With participants' consent, interviews were scheduled at a mutually convenient time and conducted in a private, quiet setting to avoid interruptions or disturbances. The interview guide comprised several key themes presented as open‐ended questions, which were addressed in a semistructured manner (Table S2).

A total of 37 individuals participated in the original study, which broadly investigated the lived experience of care and hospitalization among patients undergoing neurorehabilitation. Interviews lasted approximately 35 minutes each. For this secondary analysis, a purposive subsample of 17 interviews was independently identified by two researchers (M.D., M.V.) to ensure rich, information‐dense cases relevant to the research question and the FoC framework. In cases of uncertainty regarding the inclusion of an interview, a third researcher (C.A.C.) was consulted to reach consensus, further minimizing selection bias. Selection was guided by the completeness and relevance of the data rather than by any preconceived hypotheses.

### Data analysis

A descriptive deductive content analysis was conducted^25^ using Atlas.ti version 24, guided by the FoC framework and its core components. The process began with the identification of meaning units, followed by initial coding. The coding and categorization processes were iterative and collaborative, with ongoing discussions among research team members guiding the refinement of the coding structure and ensuring interpretative rigor. Codes were then sorted and organized in relation to the elements and dimensions of the FoC framework. The findings are presented as categories aligned with the framework components.

To facilitate a transparent and inclusive analytical process within the international research team, all quotations were translated into English.

### Rigor

Rigor in this QSA was supported through multiple strategies. A key methodological feature was that only some members of the research team conducting the secondary analysis had also been involved in the primary study. This partial continuity ensured deep familiarity with the data and the original study context, and the inclusion of researchers new to the dataset enriched interpretation by bringing fresh perspectives and alternative analytical lenses. Reflexive practices, including journaling and team discussions, together with peer debriefing, supported analytic transparency and critical interpretation. Investigator triangulation and interrater agreement further enhanced the credibility and consistency of coding, and a detailed audit trail documented all analytic decisions. As all data had been collected prior to the secondary analysis, emerging interpretations could not influence data generation, thereby minimizing researcher‐driven bias despite the presence of overlapping researchers. Analytic limitations, such as the inability to conduct follow‐up interviews, were addressed by focusing on information‐rich cases and triangulating interpretations within the research team, ensuring findings remained grounded in participants' original accounts while generating meaningful, theory‐informed insights.

### Ethical considerations

Ethical approval was granted by the Territorial Ethics Committee of the Central‐East Veneto region on April 3, 2023 (reference code EM/S.Camillo/24). The research was conducted in accordance with the ethical principles of the Declaration of Helsinki^26^ and fully complied with the General Data Protection Regulation (GDPR Regulation [EU] 2016/679), ensuring the protection of participants' rights and personal data. All participants provided written informed consent prior to participation.

Participant privacy and confidentiality were rigorously maintained. Personal data were treated with strict confidentiality, and anonymity was ensured at all stages. Findings were presented in anonymized form, and access to raw data was restricted exclusively to research team members.

## RESULTS

### Characteristics of the sample

The sample consisted of 17 individuals (9 females, 8 males) with a mean age of 63.4 years. Most participants (*N* = 16, 94.1%) had a caregiver. Patients presented with a range of neurological conditions, including stroke (*N* = 8), Parkinson disease (*N* = 5), multiple sclerosis (*N* = 3), and brain tumor (*N* = 1).

Functional independence, as measured by the Barthel Index, varied considerably. Scores at admission ranged from 0 to 80, with improvements observed in all cases by discharge (scores at discharge ranged from 35 to 95). Despite overall progress, most participants remained partially dependent at discharge, reflecting the complexity and severity of their conditions.

A detailed summary of participants' demographic and clinical characteristics is provided in Table 1.

**TABLE 1:** Characteristics of the sample (*N* = 17).

ID	Gender	Age, y	Profession	Presence of caregiver	Diagnosis	Y of illness	Previous admissions to neurorehabilitation	Barthel admission	Barthel discharge
P1	Female	57	Businessman	Yes	Brain tumor	4	No	25	45
P2	Female	56	Concierge	No	Parkinson disease	12	Yes	60	95
P3	Female	57	Lawyer	Yes	Parkinson disease	11	No	50	90
P4	Male	77	Retired	Yes	Parkinson disease	10	No	80	90
P5	Male	54	Retired	Yes	Parkinson disease	14	Yes	65	90
P6	Male	76	Retired	Yes	Parkinson disease	12	Yes	25	60
P7	Male	54	Technical expert	Yes	Multiple sclerosis	4	Yes	30	60
P8	Female	59	Teacher	Yes	Multiple sclerosis	27	Yes	30	55
P9	Female	68	Retired	Yes	Multiple sclerosis	38	Yes	30	55
P10	Female	34	Housewife	Yes	Ischemic stroke right hemisphere	Acute	‐	20	40
P11	Male	73	Retired	Yes	Ischemic stroke left hemisphere	Acute	‐	0	45
P12	Female	84	Retired	Yes	Hemorrhagic stroke left hemisphere	Acute	‐	20	40
P13	Male	85	Retired	Yes	Ischemic stroke left hemisphere	Acute	‐	25	50
P14	Male	60	Chef	Yes	Hemorrhagic stroke right hemisphere	Acute	‐	5	35
P15	Male	74	Retired	Yes	Ischemic stroke right hemisphere	Acute	‐	30	60
P16	Female	54	Housewife	Yes	Ischemic stroke right hemisphere	Acute	‐	70	90
P17	Female	55	Musician	Yes	Hemorrhagic stroke right hemisphere	Acute	‐	60	80

*Note*: Acute refers to the fact that the admission to neurorehabilitation took place after an acute hospitalization.

### Three dimensions of the FoC framework

The findings are organized according to the three core dimensions of the FoC framework (Table 2). Participants' narratives often reflect an integrated experience of care with overlaps between the physical, psychosocial, and relational dimensions both evident and unavoidable.

**TABLE 2:** Coding tree.

	Categories	Codes	*N*	Quotes
Care recipient needs	Physical	Comfort	7	*The bed is made right away in the morning in a positive way, and this is something positive that makes you feel good. (P7)*
		Personal cleansing and dressing	5	*In the morning, the health care assistants come to wash us, and for me it's a way to feel renewed. In the morning they have to change my diaper, and also at lunchtime and when putting me to bed. During the night, they come by at least four times, each time to check if I'm clean. And that, in its own way, is a small step forward. Rehabilitation has to be complete: mind, body, everything. (P1)*
		Toileting needs	4	*When I needed help, I rang the call bell, and in a moment the nurse assistants came. They come to change me and to wash me. (P7)*
		Mobility	3	*With physiotherapy, you go for a walk and, in any case, you get the body moving a little bit. (P2)*
		Rest and sleep	3	*The lady who shares my room is in constant suffering—at night, during the day, 24/7, and it's hard to hear it all the time. And it's not like she's ringing the call bell for help. All night she keeps saying ‘oh oh oh, ah ah ah’, it's hard to get any rest. (P3)*
		Medication management	2	*When the nurses give me the medicine, they stay there and tell me, “You have to take your medicine,” and they remain here until I take all of it. (P12)*
		Safety	1	*One day I fell to the floor, and within 30 s I had the nurse and the nurse assistants all around me, helping me. (P5)*
	Psychosocial	Dignity	7	*A bit of embarrassment and a bit of discomfort, yes, because, how can I put it, the colleagues who wash us are always female nurses. So there's that sense of modesty, I don't know how to say it … not shame, just a bit of modesty. (P13)*
		Communication	6	*They [health care professionals] ask me if I feel like talking. For example, today my leg hurts, and talking with others helps. (P13)*
		Privacy	5	*Since I'm in a room with two beds and I share the space with someone, there's never really a moment of privacy. (P1)*
		Respect	3	*All [health care workers] are kind and caring with us, and also very patient. (P13)*
		Emotional well‐being	3	*[Nurse] was able to do everything calmly, peacefully. With a kind of cheerfulness that she conveys. It makes everything easier, because even the patient is more willing when there's someone who listens to you, helps you, laughs with you. (P4)*
		Being involved and informed	1	*I ask: what are these medicines and what are they for? And the nurses explain it to me. And I ask: what are these, because I'd like to know what I'm taking. I only take three medicines in the evening and two in the morning for my stomach. (P12)*
Caregiver actions	Relational	Being compassionate	8	*When I came back from the hospital, they welcomed me warmly with a little cloth in front of the television, like a mother taking care of her child … which, at my age, really touched me. I was very pleasantly surprised by a kindness that wasn't expected. (P3)*
		Being empathetic	5	*In my opinion, based on their experience [health care professionals], they can immediately see and understand how a person's day is going, how it started—whether good or bad. (P2)*
		Being present	5	*Every morning she [the nursing coordinator] comes by to say, “Good morning, is everything okay?” So this is a pleasant thing, absolutely, it makes you feel her presence. (P2)*
		Active listening	2	*[The health care assistant] has skills, in my opinion, that are even more connected to the emotional side—the part about being away from home, not in your own environment. They are here to help you and to listen. (P2)*
		Supporting and involving families and carers	3	*One day I asked for permission because I went to eat something with my uncles. My uncles are almost like my mom and dad, and they authorized me. This helped me. (P7)*
		Helping care recipients to cope	1	*In 1 year, all the health care professionals really lifted my spirits, and that then impacts physical rehabilitation. (P2)*

*Note*: *N*, Number of quotations identified for the respective codes.

### Physical dimension

Participants described a range of physical care activities integral to their rehabilitation experience. Although many of these actions were perceived as routine, they were also understood as meaningful contributors to recovery and dignity.

Comfort was associated not only with physical aspects, such as a well‐made bed, but also with the sense of care and attentiveness demonstrated by health care professionals. Participants highlighted the affectionate and considerate attitude of the health care professional, which contributed positively to both their emotional wellbeing and engagement in rehabilitation.

*Whatever I ask, they [health care professionals] are very attentive to the needs of the patient*. (P4)

Such gestures, though seemingly simple, were perceived as meaningful signs of care, strengthening a therapeutic environment that fosters trust and supports recovery.

Participants highly valued personal washing and dressing, especially those requiring assistance due to incontinence. These care activities were not only associated with hygiene but were also perceived as moments of personal renewal and symbolic progress within the rehabilitation journey. Continuity of care, even during the night, was appreciated, as health care professionals regularly checked on patients' cleanliness and comfort.

*They come during the night at least four times… It's still a small step forward. Rehabilitation must be complete—mind, body, everything*. *(P7)*

Toileting assistance was also described positively, with participants noting the prompt response of health care professionals and the consistent feeling of being cared for. These intimate moments, though initially uncomfortable, eventually become normalized and fostered a sense of shared vulnerability and equality among patients.

*At first, when the health care assistant washes your intimate parts, it feels a bit strange, but eventually you understand it's normal*. *(P7)*

These experiences highlight how fundamental care tasks, when administered with respect and presence, can enhance both the physical and relational dimensions of recovery. Mobility was perceived as a fundamental component of care, particularly through physiotherapy. Given the neurological nature of their conditions, which often significantly affected motor function, participants viewed physical rehabilitation as essential for restoring autonomy and promoting bodily recovery. Engaging in movement, even in small ways, was seen as a meaningful step toward regaining independence.

*M. comes over and sees me watching TV, lying all crooked in bed with my neck twisted — and she just lifts me up, no questions asked. She sees it and straightens me out*. (P6)

Rest and sleep were often compromised by environmental factors, such as loud noise or the suffering of other patients. Sharing a room with individuals experiencing severe discomfort made it difficult for some participants to rest adequately, affecting their overall well‐being.

*So, at night when I got up to go to the bathroom, she'd see me get up and say, “Please, can you give me some water?” And I never said no—I always gave her water. Then she started asking, “Can you move my pillows?” and I said, “No, I can't do that because I have to keep my balance, so press the call button.”. But yeah, I always gave her water at night*. (P17)

The management of medication was described as attentive and supportive. Participants appreciated how nurses ensured adherence while maintaining a respectful and involved approach.

*When the nurses give me the medicine, they stay there and tell me, ‘You have to take your medicine,’ and they remain here until I take all of it*. *(P12)*

Finally, regarding safety, participants recalled episodes in which health care personnel responded rapidly to accidents or unexpected needs, reinforcing a sense of protection and close monitoring.

*One day I fell to the floor, and within 30 seconds I had the nurse and the nurse assistants all around me, helping me*. (P5)

### Psychosocial dimension

Participants described several aspects of psychosocial care as central to their rehabilitation experience. The radar chart illustrates that the key dimensions emerging from participants' accounts were dignity, communication, privacy, respect, and emotional well‐being. Dignity and communication were the most frequently and richly described aspects, indicating their central role in the psychosocial experience of care.

Dignity emerged as a nuanced and multifaceted aspect of care, particularly in relation to intimate assistance. For some participants, feelings of embarrassment arose when personal hygiene was provided by professionals of the opposite gender. Even when care was delivered with discretion and professionalism, the situation could still evoke discomfort, reflecting the delicate balance between necessary physical care and the preservation of personal dignity.

*There's that sense of modesty… the colleagues who wash us are always female nurses*. *(P13)*

For others, dignity was closely tied to being kept clean and taken care for, especially among patients experiencing incontinence and loss of autonomy. Witnessing the condition of fellow patients who struggled with expressed emotional distress due to unmet hygienic needs increased participants' awareness of the human value of cleanliness and compassion in care.

*There are these older women who soil themselves and have bedsores and cry in pain day and night. You can't just walk past. In this profession, you're here not only to treat and assist, but also to humanize the illness*. *(P3)*

Communication was viewed as a crucial element influencing participants' sense of connection with professionals and their ability to express distress or needs. Patients appreciated when health care professionals invited them to talk and listened attentively.

*They [health care professionals] ask me if I feel like talking. For example, today my leg hurts, and talking with others helps*. (P13)

Privacy was a recurrent concern, particularly among those sharing rooms. Participants reported a lack of personal space, which limited comfort, emotional expression, and rest.

*Since I'm in a room with two beds and I share the space with someone, there's never really a moment of privacy*. (P1)

The participants also spoke about the importance of being treated with respect, emphasizing the value of being recognized as a person rather than just a patient.

*All [health care workers] are kind and caring with us, and also very patient*. (P13)

Emotional well‐being was positively influenced not only by the attitude and disposition of health care professionals but also by the interpersonal dynamics in shared rooms. Participants described how qualities such as calmness, empathy, and a sense of humor on the part of nurses contributed to alleviating the emotional strain of hospitalization, fostering a more relaxed and supportive environment.

*[Nurse] was able to do everything calmly, peacefully. With a kind of cheerfulness that she conveys. It makes everything easier*. *(P4)*

Some participants also found emotional comfort in sharing the room with other patients, which provided opportunities for conversation, mutual understanding, and companionship. Being around others facing similar challenges helped reduce feelings of isolation and foster a sense of belonging.

Finally, participants highlighted the importance of being involved and informed. Some valued receiving explanations about their medications and treatment, which fostered a sense of agency and control over their health.

*I ask: what are these medicines and what are they for? And the nurses explain it to me… I'd like to know what I'm taking*. (P12)

### Relational dimension

In participants' narratives, the relational dimension of care emerged as a key component of the rehabilitation experience, significantly shaping emotional well‐being, sense of security, and motivation throughout hospitalization. Within this dimension, empathy, presence, and compassion were frequently emphasized, underscoring the importance of relational continuity and emotional closeness, which fostered trust and a sense of safety. Health care professionals who were attuned to the emotional signals of the patients were perceived as particularly supportive. Participants vividly recalled moments of compassion, particularly when gestures of kindness made them feel “at home” or genuinely cared for. These included verbal reassurance, emotional availability, and nurturing behaviors reminiscent of family affection—such as a mother caring for her child.

*No, no, they're amazing girls* [the health care assistants], *I can't say a bad word. They've got strong personalities, they're there for me, they try to give you strength, to lift you up. ‘Come on, don't give up!’ I really can't complain—they've treated me well, all of them*. *(P9)* .

Empathy was described as a distinguishing trait of health care professionals. Participants valued the ability of the health care professional to intuitively recognize their emotional states and respond with sensitivity and care.

*In my opinion, based on their experience, they can immediately see and understand how a person's day is going, how it started—whether good or bad*. *(P2)*

The consistent presence of health care professionals in daily routines also played a key relational role. Simple acts, such as morning check‐ins, were interpreted not only as organizational gestures but also as expressions of proximity and emotional support, strengthening the therapeutic relationship.

*They used to say to me, “Don't worry, S., we are here for this.”* *(P3)*

Closely connected to the sense of presence was the theme of active listening, which participants identified as central to their experience of relational care. They appreciated that health care professionals were able to attend not only to clinical aspects but also to the emotional and existential challenges of being hospitalized and away from home.

*[The health care assistant] has skills, in my opinion, that are even more connected to the emotional side—the part about being away from home… They are here to help you and to listen*. *(P2)*

Participants also underscored the importance of involving family members and caregivers, not only in care‐related decisions but also in sustaining personal relationships and social identity. Even small gestures or permissions that allowed patients to preserve connections with loved ones were deeply valued.

*Whatever, but I feel different when my wife is here. When my wife is not here, I feel bad. I am used to a family. Without the family I feel lost*. *(P15)*

Finally, relational competence was described as essential in supporting patients to cope with the psychological and physical demands of neurorehabilitation. Feeling emotionally supported by health care professional positively influenced their engagement and motivation throughout the rehabilitation process.

*In one year, all the health care professionals really lifted my spirits, and that then impacts physical rehabilitation*. *(P2)*

## DISCUSSION

This study provides an in‐depth understanding of how individuals with neurological diseases perceive their fundamental care needs during neurorehabilitation. By applying the FoC framework, it brings to light aspects of caring that are often underappreciated, underdocumented, or entirely omitted in clinical records and traditional models of care.^12^, ^13^ Core aspects identified by patients—such as relational presence, emotional attunement, and dignity‐preserving behaviors—represent what they regard as “good” caring, yet these elements frequently remain invisible within the procedural logic of contemporary health care.^27^, ^28^, ^29^

In the physical dimension, our findings underscore that dignity in care extends well beyond technical execution. Participants described basic care activities, such as hygiene, toileting, and dressing, not as mere routines but as pivotal moments of personal recognition and human connection. These acts gained meaning when carried out with empathy, attentiveness, and emotional presence, reinforcing the view that dignified care is inherently relational.^2^, ^4^, ^10^ Mobility, although clinically relevant for all participants as neurological patients, appeared less frequently in their narratives. This absence may reflect a delegation of responsibility primarily to physical therapists, suggesting a missed opportunity for nurses and health care assistants to participate more actively in the promotion of autonomy through everyday movement. In addition, within neurorehabilitation settings, mobilization is often framed as a highly specialized intervention and a core focus of rehabilitation, leading nursing staff to await explicit guidance from physical therapists. Although intended to ensure safety and expertise, this reliance may inadvertently contribute to delayed or missed responses to patients' everyday mobility needs. This interpretation aligns with calls for greater interdisciplinary collaboration in mobilization practices. Furthermore, Richards et al.^11^ highlight that the evidence on the effectiveness of nursing interventions in areas such as mobility remains limited and of low quality, restricting guidance for practice. Consistent with Crooks et al.,^30^ RNs and health care assistants perceive greater barriers to patient mobilization than physical therapists, particularly related to workload, time constraints, and competing priorities, which lead to mobility being deprioritized in daily care. Role ambiguity, limited confidence in safely mobilizing complex patients, and safety concerns further reduce nursing engagement.^30^ Together, these factors help explain why nurses and health care assistants, despite their constant bedside presence, are often underused in patient mobilization efforts and why mobility activities such as ambulation are often missed or omitted from routine care.^13^ Lastly, remaining within the physical dimension, health care assistants played a particularly visible and valued role in delivering intimate and comfort‐oriented care. Patients often described their contributions in emotionally rich terms, conveying presence, reassurance, and even maternal care. This highlights health care assistants' role as relational anchors in daily routines.^31^

In the psychosocial dimension, participants repeatedly emphasized respectful communication, emotional support, and being adequately informed as central to their well‐being. These factors were not seen as peripheral, but as integral to the very experience of care. Even seemingly small relational gestures, such as explaining the purpose of medications, were perceived as meaningful acts that restored agency and strengthened trust. This finding aligns with prior studies showing that psychosocial care contributes significantly to patient satisfaction and recovery.^1^, ^16^, ^28^ Privacy emerged as a significant yet frequently overlooked component of psychosocial care. Although participants often accepted the constraints of shared hospital rooms, many expressed a sense of unease. Privacy, autonomy, and dignity are often challenged by shared rooms and unavoidable exposure, while the sense of home, belonging, and personal routines is difficult to replicate.^32^ Dignity is supported when residents have private spaces and can keep personal items that connect them to their former homes, and it appears not as a fixed attribute but as something continuously negotiated through daily interactions between residents and staff. As Malcolm^33^ observed, patients tend to experience a conditional acceptance of privacy loss, shaped by environmental limitations, professional routines, and institutional norms. Importantly, this acceptance does not negate the emotional burden or the communicative barriers it imposes. This discomfort extended beyond personal preference, reflecting deeper relational and ethical concerns. Successful adaptation to institutional living hinges on emotional support and meaningful engagement from both staff and families, making relational and environmental support essential.^32^ Moreover, to promote privacy, autonomy, and meaningful social interactions, rehabilitation settings should be designed to resemble home environments.

In the relational dimension, participants' narratives focus on relational care that extended beyond technical competence, fostering spaces where they felt seen, supported, and emotionally sustained throughout their rehabilitation journey. Empathy, presence, and continuity emerged as critical elements of this experience. When health care professionals were attuned to patients' emotional signals, offered reassurance, or shared small moments of humanity, they established a climate of belonging, trust, and emotional safety. These findings align with Kitson et al.,^2^, ^4^ and more recent contributions^5^, ^15^ that place relational competence at the heart of person‐centered fundamental care. Participants demonstrated strong reflexivity in relational dynamics, emphasizing emotionally connected, person‐to‐person interactions as the ideal nurse‐patient relationship, in line with the relational foundations of the FoC framework. The literature shows that empathetic approaches, especially in long‐term care, support communal routines tailored to individual preferences, such as bedtime or bath time, promoting shared understanding among residents, families, and staff.^34^ Patients' and families' experiential knowledge, far from anecdotal, constitutes a vital resource for tailoring care practices to individual needs and values.^14^ Staff supported residents' autonomy and emotional needs by actively listening, remaining curious, involving families in care planning, and tailoring care to individual preferences. Health care staff should move beyond standardized care plans, relying on experiential knowledge and flexible approaches to provide personalized care that fosters mutual trust and meaningful connections.^34^ Recognizing patients as active informants rather than passive recipients is thus essential for achieving truly person‐centered care.

Finally, this study illustrates the value of the FoC framework as a guiding tool in neurorehabilitation. By drawing attention to the dimensions of care that patients identify as most meaningful, it supports nursing and interdisciplinary teams, including health care assistants, in addressing not only physical recovery but also emotional security, human dignity, and relational continuity. This is consistent with the findings of Kitson et al.,^2^, ^4^ who showed that stroke survivors' experiences of care are profoundly shaped by the integration of physical, emotional, and relational needs.

### Strengths and limitations of the work

This study contributes original insights into how individuals with neurological disorders perceive their fundamental care needs within neurorehabilitation. A notable strength is the use of the FoC framework, which provided a robust theoretical lens to interpret patients' narratives and capture the multidimensional nature of care. The original dataset was suitable for a FoC‐informed reanalysis because interviews provided detailed accounts of relational, psychosocial, and physiological aspects of care. The inclusion of rich first‐person accounts from a diverse group of patients strengthened the depth of the analysis and underscored the importance of relational and emotional aspects of care, often underrepresented in quantitative or task‐focused research.

This study has several limitations. First, as a QSA, the dataset was originally collected for a broader project rather than specifically designed around the FoC framework. As a result, the data did not fully align with the new research question, which may have affected the relevance, completeness, and specificity of the findings. The analysis prioritized depth over breadth, focusing on cases and excerpts that were particularly information rich. Second, the sample was purposively selected from a single neurorehabilitation hospital in Italy. Although the narratives were rich in detail, the relatively small and localized nature of the sample restricts the transferability of the findings to wider neurological populations or other health care contexts. Third, the exclusive focus on patients' perspectives, without triangulation with the views of nurses, health care assistants, or other professionals, inhibits a more comprehensive understanding of the dynamics of fundamental care. Although participants frequently highlighted the role of health care assistants, their contributions were not the explicit focus of the theoretical analysis, pointing to the need for future research to explore this aspect more systematically. Finally, the findings reflect an integrated and often overlapping patient experience of care. In line with previous literature, the physical, psychosocial, and relational dimensions of care were deeply interlaced, making it challenging to disentangle them without oversimplifying the narratives. Although this interconnectedness is inherent to person‐centered care, it poses interpretive challenges when applying structured analytical frameworks such as the FoC.

### Implications for practice

This study highlights several important implications for nursing practice, particularly within neurorehabilitation settings where patients face complex, long‐term, and multidimensional care needs. Based on our findings, we propose the following practice‐oriented recommendations:

1. Incorporate the FoC framework into the delivery of nursing care: Its systematic application can support nurses in identifying and integrating physical, psychosocial, and relational needs, thereby ensuring that care remains aligned with what matters most to patients. This point could be achieved through targeted training and certain modifications to care delivery models (eg, adapting documentation).
2. Improve collaboration among nurses, health care assistants, and physical therapists: Patients frequently described health care assistants as highly present and emotionally supportive figures in their daily care routines. To fully acknowledge and harness the value of this role, it is essential to ensure appropriate training, supervision, and coordination by RNs. Moreover, encourage interdisciplinary collaboration so that mobility is consistently supported as part of routine care, rather than delegated solely to physical therapists.
3. Prioritize patient dignity and autonomy: Provide private spaces whenever possible, allow personal items from home, and respect resident routines and preferences. Recognize that dignity is continuously negotiated through interactions. Enhance shared spaces, such as indoor and outdoor common areas, to support social interaction.
4. Promote emotional awareness and incorporate the experiential knowledge of patients and families: Staff should actively listen, respond to patients' emotional cues, and involve patients and families in care planning and decision‐making. Treat their insights as essential for tailoring care to individual needs and preferences, and recognize that even small gestures, such as explaining what is happening, offering reassurance, or showing empathy, can significantly enhance trust, engagement, and overall satisfaction.

Finally, our findings reaffirm the centrality of the nurse–patient relationship in fundamental care. The “how” of care, that is empathy, presence, attention, and relational continuity, matters as much as the “what.”

### Recommendations for further research

Future studies should expand investigation of the FoC framework in neurorehabilitation by incorporating multiple perspectives, including those of nurses, health care assistants, and family caregivers, to gain a more comprehensive understanding of care dynamics. Comparative studies across different rehabilitation contexts, health care systems, and cultural settings would strengthen the transferability of findings. In addition, dedicated research on the role of health care assistants is warranted, given their prominence in patients' accounts as providers of comfort and relational support. Such studies could inform workforce development, training, and interprofessional collaboration, ultimately advancing the quality and person‐centeredness of fundamental care in neurorehabilitation.

## CONCLUSIONS

This study reinforces the relevance of the FoC framework within neurorehabilitation settings by capturing patients' lived experiences of fundamental care. The findings highlight the need to move beyond task‐oriented or procedural conceptions of nursing. Care should preserve dignity and autonomy, involve patients and families, and be delivered in home‐like environments. Treating care activities as relational opportunities, with consistent staff presence, emotional attunement, and interdisciplinary collaboration, ensures care is personalized, meaningful, and aligned with patients' priorities. The FoC framework offers a multidimensional and integrated lens that links physical, psychosocial, and relational needs within a cohesive, person‐centered, and context‐aware model of care.

## FUNDING INFORMATION

No external funding.

## ETHICS STATEMENT

Ethical approval was granted by the Territorial Ethics Committee of the Central‐East Veneto region on April 3, 2023 (reference code EM/S.Camillo/24).

## DISCLOSURE

The authors declare no conflicts of interests.

## PATIENT CONSENT STATEMENT

All participants provided written informed consent prior to participation.

## Supporting information


**Table S1.** Principal characteristics of registered nurses (RNs) and health care assistants.
**Table S2.** Interview questions.

## Data Availability

The data that support the findings of this study are available on request from the corresponding author. The data are not publicly available due to privacy or ethical restrictions.

## References

[1] van Belle E , Giesen J , Conroy T , et al. Exploring person‐centred fundamental nursing care in hospital wards: a multi‐site ethnography. J Clin Nurs. 2020;29(11–12):1933‐1944. doi:10.1111/jocn.15024 PMC7319433 31408557

[2] Kitson A , Conroy T , Kuluski K , Locock L , Lyons R . Reclaiming and redefining the Fundamentals of Care: Nursing's response to meeting patients' basic human needs. 2013.

[3] Kitson A , Conroy T , Wengstrom Y , Profetto‐McGrath J , Robertson‐Malt S . Defining the fundamentals of care. Int J Nurs Pract. 2010;16(4):423‐434. doi:10.1111/j.1440-172X.2010.01861.x 20649678

[4] Kitson A , Dow C , Calabrese J , Locock L , Athlin Å . Stroke survivors' experiences of the fundamentals of care: a qualitative analysis. Int J Nurs Stud. 2013;50(3):392‐403. doi:10.1016/j.ijnurstu.2012.09.017 23083623

[5] Muntlin Å , Jangland E , Laugesen B , et al. Bedside nurses' perspective on the fundamentals of care framework and its application in clinical practice: a multi‐site focus group interview study. Int J Nurs Stud. 2023;145:104526. doi:10.1016/j.ijnurstu.2023.104526 37390582

[6] Pene BJ , Aspinall C , Komene E , et al. The Fundamentals of Care in practice: a qualitative contextual inquiry. Nurs Inq. 2025;32(2):e70000. 10.1111/nin.70000 PMC11873673 40025840

[7] Mudd RNA , Feo R , Conroy T , Kitson A . Where and how does fundamental care fit within seminal nursing theories: a narrative review and synthesis of key nursing concepts. J Clin Nurs. 2020;29(19–20):3652‐3666. doi:10.1111/jocn.15420 PMC7540068 33463848

[8] Ottonello G , Napolitano F , Musio ME , et al. Fundamental care: an evolutionary concept analysis. J Adv Nurs. 2023;79(6):2070‐2080. doi:10.1111/jan.15451 36226779

[9] Kaukkila HS , Peltonen LM , Korteniemi AS , et al. The quality of fundamental care as perceived by nurses and patients in a hospital setting: a descriptive comparative study. Nurs Open. 2025;12(4):e70234. doi:10.1002/nop2.70234 PMC12014395 40263644

[10] Feo R , Frensham LJ , Conroy T , Kitson A . “It's just common sense”: preconceptions and myths regarding fundamental care. Nurse Educ Pract. 2019;36:82‐84. doi:10.1016/j.nepr.2019.03.006 30889468

[11] Richards D , Hilli A , Pentecost C , Goodwin V , Frost J . Fundamental nursing care: a systematic review of the evidence on the effect of nursing care interventions for nutrition, elimination, mobility and hygiene. J Clin Nurs. 2018;27(11–12):2179‐2188. doi:10.1111/jocn.14150 PMC6001513 29156087

[12] Feo R , Conroy T , Alderman J , Kitson A . Implementing fundamental care in clinical practice. Nurs Stand. 2017;31(32):52‐62. doi:10.7748/ns.2017.e10765 28378679

[13] Danielis M , Fantini M , Sbrugnera S , et al. Missed nursing care in a long‐term rehabilitation setting: findings from a cross‐sectional study. Contemp Nurse. 2021;57(6):407‐421. doi:10.1080/10376178.2022.2029515 35023449

[14] Danielis M , Zanotti R . ChatGPT answers a frequently asked question about nursing: what it is and what it is not. Nurs Inq. 2024;31(1):e12620. doi:10.1111/nin.12620 38149469

[15] Savoie C , Rey S , Yokota S , et al. Fundamental Care's state of knowledge around the world: where are we now? A scoping review. J Adv Nurs. 2023;79(3):865‐884. doi:10.1111/jan.15278 35511714

[16] Collier A , De Bellis A , Hosie A , et al. Fundamental care for people with cognitive impairment in the hospital setting: a study combining positive organisational scholarship and video‐reflexive ethnography. J Clin Nurs. 2020;29(11–12):1957‐1967. doi:10.1111/jocn.15056 31495005

[17] Mandal L , Seethalakshmi A , Rajendrababu A . Rationing of nursing care, a deviation from holistic nursing: a systematic review. Nurs Philos. 2020;21(1):e12257. doi:10.1111/nup.12257 31429179

[18] Recio‐Saucedo A , Dall'Ora C , Maruotti A , et al. What impact does nursing care left undone have on patient outcomes? Review of the literature. J Clin Nurs. 2018;27(11–12):2248‐2259. doi:10.1111/jocn.14058 PMC6001747 28859254

[19] Feo R , Kitson A , Conroy T . How fundamental aspects of nursing care are defined in the literature: a scoping review. J Clin Nurs. 2018;27(11–12):2189‐2229. doi:10.1111/jocn.14313 29514402

[20] Hampel H , Gao P , Cummings J , et al. The foundation and architecture of precision medicine in neurology and psychiatry. Trends Neurosci. 2023;46(3):176‐198. doi:10.1016/j.tins.2022.12.004 PMC10720395 36642626

[21] Mekkes N , Groot M , Hoekstra E , et al. Identification of clinical disease trajectories in neurodegenerative disorders with natural language processing. Nat Med. 2024;30(4):1143‐1153. doi:10.1038/s41591-024-02843-9 PMC11031398 38472295

[22] Tate JA , Happ MB . Qualitative secondary analysis: a case exemplar. J Pediatr Health Care. 2018;32(3):308‐312. doi:10.1016/j.pedhc.2017.09.007 PMC5911239 29254902

[23] Heaton J . Secondary analysis of qualitative data: an overview. Hist Soc Res. 2008;33(3):33‐45.

[24] Tong A , Sainsbury P , Craig J . Consolidated criteria for reporting qualitative research (COREQ): a 32‐item checklist for interviews and focus groups. International J Qual Health Care. 2007;19(6):349‐357. doi:10.1093/intqhc/mzm042 17872937

[25] Elo S , Kyngäs H . The qualitative content analysis process. J Adv Nurs. 2008;62(1):107‐115. doi:10.1111/j.1365-2648.2007.04569.x 18352969

[26] World Medical Association . Declaration of Helsinki: Ethical Principles for Medical Research involving human subjects. 2024. Retrieved December 20, 2024 from https://www.wma.net/what-we-do/medical-ethics/declaration-of-helsinki/) 10.1001/jama.2013.281053 24141714

[27] Chukwuere P . Connecting and attuning mental health Care for Adolescent Patients: the role of presence practice. SAGE Open Nurs. 2023;9:23779608231193758. doi:10.1177/23779608231193758 PMC10424542 37584035

[28] Wensley C , Botti M , McKillop A , Merry AF . Maximising comfort: how do patients describe the care that matters? A two‐stage qualitative descriptive study to develop a quality improvement framework for comfort‐related care in inpatient settings. BMJ Open. 2020;10(5):e033336. doi:10.1136/bmjopen-2019-033336 PMC7239554 32430447

[29] Ziebland S , Locock L , Fitzpatrick R , et al. Informing the development of NICE (National Institute for Health and Care Excellence) quality standards through secondary analysis of qualitative narrative interviews on patients' experiences. 2014 https://www.ncbi.nlm.nih.gov/books/NBK263660/ 25642541

[30] Crooks E , Rampley T , Weeks DL , Billings C , Stengem D , Rangel T . Perceived barriers to patient mobilization among therapy and nursing acute care staff: a multi‐site survey study. Arch Phys Med Rehabil. 2024;105(2):243‐250. doi:10.1016/j.apmr.2023.06.018 37429536

[31] Campbell A , Layne D , Scott E . Relational quality of registered nurses and nursing assistants: influence on patient safety culture. Healthcare (Basel). 2021;9(2):189. doi:10.3390/healthcare9020189 PMC7916158 33572352

[32] Xue DM , Wang DN , Bian Y . Dignity of older adults in long‐term care facilities: a systematic review of qualitative evidence from residents, staff, and relatives. Healthcare (Basel). 2025;13(22):2839. doi:10.3390/healthcare13222839 PMC12652313 41302227

[33] Malcolm H . Does privacy matter? Former patients discuss their perceptions of privacy in shared hospital rooms. Nurs Ethics. 2005;12(2):156‐166. doi:10.1191/0969733005ne772oa 15791785

[34] Gurung S , Chaudhury H . Relationship‐centered Care for Older Adults in long‐term care homes: a scoping review. J Appl Gerontol. 2025;44(9):1513‐1532. doi:10.1177/07334648241309761 PMC12335636 39787049

