# Supplementary material for: How do neurorehabilitation patients perceive their fundamental care needs? Findings from a qualitative study
Source: PM R. 2026 Oct 15;18(11):1284–96. doi: 10.1002/pmrj.70236 (PMC13768722; doi:10.1002/pmrj.70236)
Supplement: Supplementary file 1 [file PMRJ-18-1284-s001.docx]

| **Professional figure** | **Characteristics of study program** |
| --- | --- |
| Registered nurses | Since 2003, all Registered Nurses have been educated at the university level, obtaining a three-year Bachelor of Nursing degree (5,400 hours, in line with European Directives). Before this reform, nursing education was primarily vocational and concluded with a regionally awarded Nursing Diploma.  Since 2004, nurses aiming for advanced managerial or clinical roles have been able to pursue a Master of Nursing Science degree. However, no Master’s degrees have been offered in clinical specialties- including rehabilitation- so expertise in these areas is largely acquired through continuing professional development programs. These programs are mandatory for all university-educated healthcare professionals and require at least 50 credits per year. |
| Healthcare assistants | Healthcare assistants receive training via regional programs that span one year (totalling 1,000 hours), focusing on vocational education. Their skills encompass delivering basic nursing care, which must be carried out under the guidance and accountability of RNs and in alignment with the predetermined patient care plan. |

**SUPPLEMENTARY TABLE 1.** Principal characteristics of Registered Nurses (RNs) and Healthcare assistants.

**SUPPLEMENTARY TABLE 2.** Interview’s questions

| 1 | Can you tell me the reason for your admission to this hospital? |
| --- | --- |
| 2 | Can you describe a typical day for you? |
| 3 | How would you describe your hospitalization experience? |
| 4 | What has been your experience with the care provided? |
| 5 | What has been your experience with rehabilitation? |
| 6 | Does the experience meet your expectations? |
| 7 | Can you describe any particularly meaningful events from this experience? |
